# Finite-Time Stochastic Stability Analysis of Permanent Magnet Synchronous Motors with Noise Perturbation

**DOI:** 10.3390/e24060791

**Published:** 2022-06-06

**Authors:** Caoyuan Ma, Hongjun Shi, Pingping Nie, Jiaming Wu

**Affiliations:** 1 School of Electrical and Power Engineering, China University of Mining and Technology, Xuzhou 221116, China; mcaoyuan@cumt.edu.cn; 2 School of Mathematics, China University of Mining and Technology, Xuzhou 221116, China; ppniecumt@outlook.com (P.N.); jiamingwu@cumt.edu.cn (J.W.)

**Keywords:** finite time, noise perturbation, permanent magnet synchronous motor, adaptive control

## Abstract

In this paper, we study the finite-time stability of permanent magnet synchronous motors (PMSMs) with noise perturbation. To eliminate the chaos in a PMSM and allow it to reach a steady state more quickly within a finite time, we propose a novel adaptive controller based on finite-time control theory. Finite-time stability implies optimal convergence time and better robustness. Finally, numerical simulations are performed to demonstrate the effectiveness and feasibility of our new results.

## 1. Introduction

Since the American meteorologist Edward Lorenz discussed chaotic phenomena in 1963 [1], chaotic behavior has been widely studied in many areas such as robots, hard disk drives, food webs, electrical power grids, secure communication and others [2,3,4,5,6,7]. As a typical nonlinear dynamical system, it is sensitive to initial conditions, parameter variables and environmental noise, and has become a common concept, enabling us to understand rich dynamic behavior. Over the past few decades, chaos control and synchronization have been intensively studied in various fields, including information processing, salt-water oscillators, semiconductor lasers, biological systems, chemical reactions and power electronics [8,9,10,11,12,13,14,15,16,17,18,19,20,21].

As is well known, PMSMs have been widely utilized due to their simple structure, low maintenance cost and inertia, high power density and efficiency. However, Hemati discovered chaos phenomena in the open-loop system of the permanent magnet motor in the mid-1990s [22]. The oscillation or irregular movement caused by chaotic behaviors can, in extreme circumstances, lead to the collapse of the systems [23]. Therefore, chaos control, aiming at eliminating the undesired chaotic behavior, has become an important nonlinear control problem [24,25,26,27,28,29]. A common method such as feedback sliding mode control is usually used for systems with parameter uncertainty and disturbance, which requires that the bounds of the uncertainty and disturbance must be known in advance. However, in practice, it is difficult to obtain these boundaries in advance. To overcome this problem, adaptive control technology is introduced into the controller design, which can estimate these unknown bounds according to a designed adaptive update law. Based on sliding mode control theory, Harb proposed a sliding mode adaptive controller to eliminate chaotic behavior in PMSMs [24]. Both Choi and Maeng explored the adaptive control of a chaotic PMSM [25,26]. Loria developed a robust linear control for chaotic PMSMs with uncertainties and further extended adaptive linear control in PMSMs [27].

In an automatic control system, the variation laws for the many inevitable random disturbances cannot be described by exact functions, but they can be expressed as noise perturbations to some extent. Taking these random disturbances into account can further improve the effectiveness and accuracy when analyzing the system control. For many common control methods, the time to achieve system stability may be infinite. However, researchers wish to make the system stable within a finite time [29,30,31,32,33]. In [29], a feedback finite-time controller was designed for achieving synchronization between two coupled networks with time-varying delays. Using adaptive state feedback controllers, Zhang et al. investigated the finite-time synchronization of discontinuous neural networks with delays and mismatched parameters [30]. Via a quantized controller, Yang et al. investigated the finite-time stabilization of switched dynamical networks with quantized coupling [31].

To the best of our knowledge, there are few results on the finite-time stability of PMSMs with noise perturbation. The difficulty in studying the stability of PMSMs with noise perturbation lies in the problems of how to construct noise coupling reasonably in the system modeling and how to use a strict mathematical method to prove the effectiveness of the control method. Motivated by the above analysis, we propose an adaptive controller based on the finite-time control theory of stochastic differential equations and the adaptive control method, to realize the stochastic finite-time stability of PMSMs.

The main innovations of this paper are as follows:

(1) The effect of noise perturbation on the finite-time stability of PMSMs is considered for the first time.
(2) Combining the advantages of the adaptive method and finite-time control technology, the designed controllers can realize the stochastic stability of the PMSM system within a finite time.
(3) We consider the effect of a control parameter α and noise on the stability, and find that there is an optimal parameter α such that the convergence time is shortest.

The highlight of this paper is that it reveals that noise perturbation within certain limits is helpful for realizing the finite-time stochastic stability of PMSMs, which is counterintuitive.

The rest of this paper is organized as follows. In Section 2, we introduce the model description and the problem formulation. In Section 3, we discuss the stochastic finite-time stability of permanent magnet synchronous motors with adaptive control. In Section 4, an illustrative example and simulations are provided to demonstrate the effectiveness and feasibility of the analytical results. Finally, in Section 5 we give some conclusions.

## 2. Model Description and Problem Formulation

**Figure 1:**
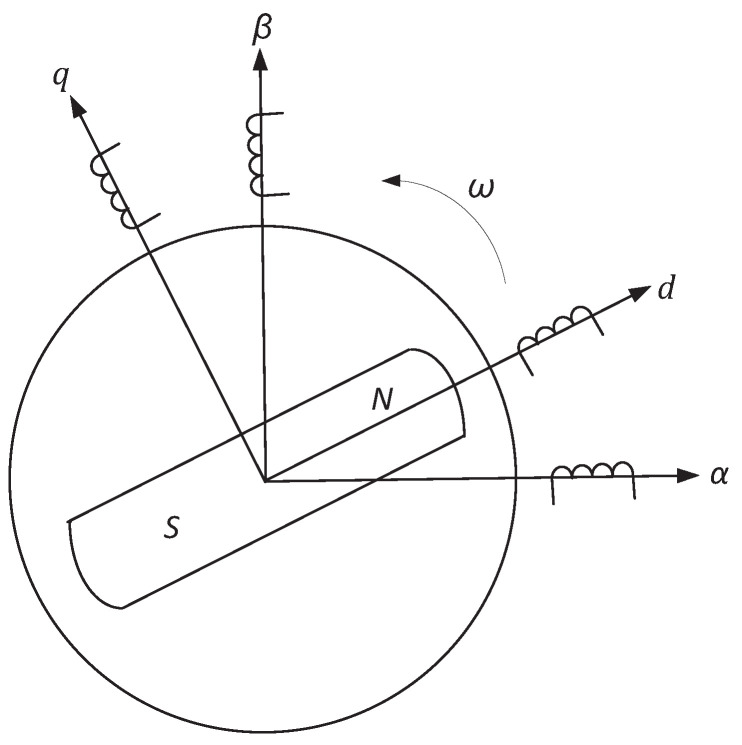
The relative position relationship between α–β axis system and *d*–*q* axis system.

In Ref [34], the mathematical model of the permanent magnet synchronous motor was first derived, and the dynamic characteristics were studied. As shown in Figure 1, an α–β axis system can rotate to a *d*–*q* axis system via the Park transformation. Using specific affine transformation and time-scale transformation, the dynamic model of a permanent synchronous motor with a smooth air gap can be described by the following dimensionless differential equations [25]:

(1) $$\begin{array}{cc} \frac{di_{d}}{dt} & =-i_{d}+i_{q}\omega +{\tilde{u}}_{d},\\ \frac{di_{q}}{dt} & =-i_{q}-i_{d}\omega +\gamma \omega +{\tilde{u}}_{q},\\ \frac{d\omega }{dt} & =\sigma \left(i_{q}-\omega \right)-{\tilde{T}}_{L}, \end{array}$$

where $i_{d}$, $i_{q}$ and ω are the state variables denoting the *d*-axis and *q*-axis stator current and angle speed of the motor, respectively, ${\tilde{u}}_{d}$ and ${\tilde{u}}_{q}$ are the *d*-axis and *q*-axis stator voltages, respectively, ${\tilde{T}}_{L}$ is the external load torque and σ>0 and γ>0 are the system operating parameters.

The external inputs ${\tilde{u}}_{d}$, ${\tilde{u}}_{q}$ and ${\tilde{T}}_{L}$ are set to zero after a given operating period of the system. Then, the unforced system (1) becomes

(2) $$\begin{array}{cc} \frac{di_{d}}{dt} & =-i_{d}+i_{q}\omega ,\\ \frac{di_{q}}{dt} & =-i_{q}-i_{d}\omega +\gamma \omega ,\\ \frac{d\omega }{dt} & =\sigma \left(i_{q}-\omega \right). \end{array}$$

Choosing specific parameters and working conditions such as σ=5.46, γ=20, $(i_{d}\left(0\right),\;i_{q}\left(0\right),\;\omega \left(0\right))=\left(5,1,-1\right)$, leads to chaotic behavior in the PMSM model (2). In order to eliminate undesirable chaos and achieve stability in a finite time, we investigate finite-time chaos control in PMSMs. Some required definitions and lemmas are given below.

**Definition** **1** ([35]). *Consider the following nonlinear dynamical system:*

(3) $$\dot{x}=f\left(x\right),$$

*where the system state variable $x\in R^{n}$, f(·) is a smooth nonlinear vector function. If there exists a constant T>0 (may depend on the initial system state $x_{0}$), such that:*

$$\lim_{t\to T}∥x\left(t\right)∥=0,$$

* and ∥x(t)∥≡0, ∀t≥T, then the system $\dot{x}=f\left(x\right)$ is finite-time stable.*

Consider the following *n*-dimensional stochastic differential equation:

(4) $$dx=\hat{f}\left(x\right)dt+\hat{g}\left(x\right)dW\left(t\right),$$

where $x\in R^{n}$ is the state vector, $\hat{f}:R^{n}\to R^{n}$ and $\hat{g}:R^{n}\to R^{n\times m}$ are continuous and satisfy $\hat{f}\left(0\right)=0,\;\hat{g}\left(0\right)=0$ and the noisy intensity matrix $W(t)={\left(w_{1},\cdots ,w_{m}\right)}^{T}$ is an *m*-dimensional Brownian motion defined on a complete probability space (Ω,F,P) with a natural filtration ${\left\{F_{t}\right\}}_{t\geq 0}$. It is supposed that Equation (4) has a unique and global solution denoted by $x\left(t,x_{0}\right)\left(0\leq t<+\infty \right),$ where $x_{0}$ is the initial state of (4).

For each $V\in C^{2,1}\left(R^{n}\times R_{+},R_{+}\right)$, the operator LV associated with Equation (4) is defined as:

(5) $$LV=\frac{\partial V}{\partial x}\cdot \hat{f}+\frac{1}{2}\mathrm{trace}\left[{\hat{g}}^{T}\cdot \frac{\partial^{2}V}{\partial x^{2}}\cdot \hat{g}\right],$$

where $\frac{\partial V}{\partial x}=\left(\frac{\partial V}{\partial x_{1}},\ldots ,\frac{\partial V}{\partial x_{n}}\right),\frac{\partial^{2}V}{\partial x^{2}}={\left(\frac{\partial^{2}V}{\partial x_{i}\partial x_{j}}\right)}_{n\times n}$.

**Definition** **2.** *For system (4), if there exists a stochastic settling time $K_{0}\left(x_{0}\right)$, such that:*

$$P\{\|x\left(t,x_{0}\right)\left\|=0\right\}=1,\forall t\geq K_{0}\left(x_{0}\right).$$

 *then the stochastic system (4) is said to achieve stochastic finite-time stability.*

**Lemma** **1** ([36]). *Assume that system (4) has a unique global solution. If there exists a positive, definite, twice continuously differentiable and radially unbounded Lyapunov function $V:R^{n}\to R^{+}$ and real numbers k>0 and 0<ρ<1, such that*

$$LV\left(x\right)\leq -k{\left(V\left(x\right)\right)}^{\rho },$$

 *then the origin of system (4) is globally stochastically finite-time stable, and $E\left[K_{0}\left(x_{0}\right)\right]\leq \frac{{\left(V\left(x_{0}\right)\right)}^{1-\rho }}{k(1-\rho )}.$*

**Lemma** **2** ([37]). *If $x_{1},x_{2},\cdots ,x_{N}>0$, then*

$$\sum_{i=1}^{N}x_{i}^{\eta }\geq {\left(\sum_{i=1}^{N}x_{i}\right)}^{\eta },0<\eta \leq 1,$$

$$\sum_{i=1}^{N}x_{i}^{\eta }\geq N^{1-\eta }{\left(\sum_{i=1}^{N}x_{i}\right)}^{\eta },\eta >1.$$

## 3. Main Results

First, we implement the noise term and controllers $u_{1},u_{2},u_{3}$ in system (2). Then, the controlled system can be described as

(6) $$\begin{array}{cc} \frac{di_{d}}{dt} & =-i_{d}+i_{q}\omega +\eta_{1}\left(i_{d}\right)\xi \left(t\right)+u_{1},\\ \frac{di_{q}}{dt} & =-i_{q}-i_{d}\omega +\gamma \omega +\eta_{2}\left(i_{q}\right)\xi \left(t\right)+u_{2},\\ \frac{d\omega }{dt} & =\sigma \left(i_{q}-\omega \right)+\eta_{3}\left(\omega \right)\xi \left(t\right)+u_{3}, \end{array}$$

where $\xi \left(t\right)=\dot{W}\left(t\right)$, and $\eta_{1}\left(\cdot \right),\eta_{2}\left(\cdot \right)$ and $\eta_{3}\left(\cdot \right)$ are simple linear functions of $i_{d},i_{q}$ and ω, respectively. Let $\eta_{1}^{2}\left(i_{d}\right)\leq 2L_{1}i_{d}^{2}$, $\eta_{2}^{2}\left(i_{q}\right)\leq 2L_{2}i_{q}^{2}$, $\eta_{3}^{2}\left(\omega \right)\leq 2L_{3}\omega^{2}$.

In order to realize the global stability of the above PMSM system, based on the theory of finite-time stability, the appropriate adaptive controllers $u_{1},u_{2},$ and $u_{3}$ are designed as follows:

(7) $$\begin{array}{cc} u_{1} & =-k_{1}i_{d}^{\alpha },\\ u_{2} & =-k_{2}i_{q}^{\alpha },\\ u_{3} & =-\sigma i_{q}-k_{3}\omega^{\alpha }, \end{array}$$

where $\alpha =\frac{p}{h}$, *p* and *h* are two positive odd integers satisfying p<h. The positive tuning parameters $k_{1},k_{2}$ and $k_{3}$ can be updated by

(8) $$\begin{array}{c} {\dot{k}}_{1}=i_{d}^{\alpha +1}-{\left(k_{1}-g_{1}\right)}^{\alpha },\\ {\dot{k}}_{2}=i_{q}^{\alpha +1}-{\left(k_{2}-g_{2}\right)}^{\alpha },\\ {\dot{k}}_{3}=\omega^{\alpha +1}-{\left(k_{3}-g_{3}\right)}^{\alpha }, \end{array}$$

where $g_{1}>0,g_{2}>0$ and $g_{3}>0$ are arbitrarily chosen constants.

**Remark** **1.** *There are many different parameters in this paper. When the operating parameters σ and γ take some special values, the system will show chaotic behavior. The control parameter α in (7) and the noise intensity λ in the numerical simulation can affect the stability of the system. In the next section, we further explore the effect of the control parameter α on the stability of the system with different noise intensities λ and with σ=5.46,γ=20.*

**Theorem** **1.** *If $L_{1},L_{2}<1;L_{3}<\sigma$, then the PMSM system (6) with noise perturbation can realize globally stochastically finite-time stability under the adaptive controllers (7).*

**Proof.**  The control process of the system is divided into two stages.

Firstly, we prove that the third subsystem in (6) can become stable in a finite time. Choose a Lyapunov candidate function as follows:

(9) $$V_{1}\left(x\right)=\frac{1}{2}\omega^{2}+\frac{1}{2}{\left(k_{3}-g_{3}\right)}^{2},$$

where $x\left(t\right)={\left(i_{d},i_{q},\omega \right)}^{T}$.

Employing Itô’s formula, one has

(10) $$\begin{array}{c} LV_{1}=\omega \dot{\omega }+\left(k_{3}-g_{3}\right){\dot{k}}_{3}+\frac{1}{2}\eta_{3}^{2}\left(\omega \right)\;\\ \;\;\;\;\;\;\;\;\;\;\;\;\;=\omega \left[\sigma \left(i_{q}-\omega \right)+u_{3}\right]+\left(k_{3}-g_{3}\right){\dot{k}}_{3}+\frac{1}{2}\eta_{3}^{2}\left(\omega \right). \end{array}$$

By adopting the designed controller $u_{3}$ of the system in (7) and the corresponding updating law of the third subsystem in (8), we obtain

LV1=ω[σ(iq−ω)−σiq−k3ωα]+(k3−g3)[ωα+1−(k3−g3)α]+12η32(ω)=−σω2−g3ωα+1−(k3−g3)α+1+12η32(ω)≤−(σ−L3)ω2−g3ωα+1−(k3−g3)α+1≤−g3ωα+1−(k3−g3)α+1=−2(α+1)2g3(12ω2)(α+1)2−2(α+1)2(12(k3−g3)2)(α+1)2≤−m1[(12ω2)(α+1)2+(12(k3−g3)2)(α+1)2],

where m1=min{2(α+1)2g3,2(α+1)2}.

Note that 12<(α+1)2<1, and it follows from Lemma 2 that

(11) LV1≤−m1[12ω2+12(k3−g3)2](α+1)2=−m1V1(α+1)2.

From Lemma 1, the third subsystem in (6) is stable in a finite time $T_{1}$, and

(12) E(T1)≤2V1(1−α)2(x0)m1(1−α),

which means that ω=0 a.s. and $k_{3}\equiv g_{3}$ when $t\geq T_{1}$.

In the second stage, when $t\geq T_{1}$, we can obtain the following subsystem:

(13) $$\begin{array}{c} \frac{di_{d}}{dt}=-i_{d}+\eta_{1}\left(i_{d}\right)\xi \left(t\right)+u_{1},\;\\ \frac{di_{q}}{dt}=-i_{q}+\eta_{2}\left(i_{q}\right)\xi \left(t\right)+u_{2}. \end{array}$$

Then, we select the following Lyapunov candidate function:

(14) $$V_{2}\left(x\right)=\frac{1}{2}i_{d}^{2}+\frac{1}{2}i_{q}^{2}+\frac{1}{2}{\left(k_{1}-g_{1}\right)}^{2}+\frac{1}{2}{\left(k_{2}-g_{2}\right)}^{2}.$$

Employing Itô’s formula, one has

LV2=id[−id+u1]+iq[−iq+u2]+(k1−g1)[idα+1−(k1−g1)α]+(k2−g2)[iqα+1−(k2−g2)α]+12η12(id)+12η22(iq)=−id2−g1idα+1−iq2−g2iqα+1−(k1−g1)α+1−(k2−g2)α+1+12η12(id)+12η22(iq)≤−id2+L1id2−g1idα+1−iq2+L2iq2−g2idα+1−(k1−g1)α+1−(k2−g2)α+1=(L1−1)id2+(L2−1)iq2−g1idα+1−g2idα+1−(k1−g1)α+1−(k2−g2)α+1≤−g1idα+1−g2idα+1−(k1−g1)α+1−(k2−g2)α+1=−2(α+1)2g1(12id2)(α+1)2−2(α+1)2g2(12iq2)(α+1)2−2(α+1)2[12(k1−g1)2](α+1)2−2(α+1)2[12(k2−g2)2](α+1)2≤−m2[(12id2)(α+1)2+(12(k1−g1)2)(α+1)2+(12iq2)(α+1)2+(12(k2−g2)2)(α+1)2],

where m2=min{2(α+1)2g1,2(α+1)2g2,2(α+1)2}.

From Lemma 2, we have

(15) LV2≤−m2[12id2+12iq2+12(k1−g1)2+12(k2−g2)2](α+1)2=−m2V2(α+1)2

Therefore, from Lemma 1 it can be seen that $i_{d}$ and $i_{q}$ are stable in a finite time $T_{2}$, and

(16) E(T2)≤2V2(1−α)2(x(T1))m2(1−α),

which means that $P\{\|x\left(t,x_{0}\right)\|=0\}=1$, when $t\geq T_{1}+T_{2}$.

Thus, by employing the adaptive controllers (7), the PMSM can achieve stochastically finite-time stability within the stochastic settling time $T_{1}+T_{2}$.

The proof is completed. □

**Remark** **2.** *From Itô’s formula, we can see that the decay rate of the function V(x) depends on the quality of LV. Hence, the convergence rate is also dominated by the quality of LV. The inequalities (11) and (15) indicate that the convergence rate of the proposed algorithm is closely related to the protocol parameters $g_{1},g_{2},g_{3}$ and α.*

**Remark** **3.** *From Equations (12) and (16), we can see that the upper bounds of the stochastic settling time $T_{1}$ and $T_{2}$ are closely related to the protocol parameter α. In the next numerical simulation, we will further explore the optimal value of control parameter α, to make the system stable as quickly as possible.*

To reveal the idea behind this paper, we describe the adaptive finite-time control design algorithm as follows:

Step 1 The initial state of the PMSM system and the input parameters of the noise intensity are determined, and the appropriate control parameter α (0<α<1) for the controllers (7) is selected to speed up the convergence process.
Step 2 Calculate the tuning parameters $k_{1},k_{2},k_{3}$ according to the updated Equation (8) for the terminal attractor and the current state of the PMSM.
Step 3 The values of $k_{1},k_{2}$ and $k_{3}$ are substituted into the controllers (7), and thus the values of $u_{1},u_{2}$ and $u_{3}$ from the adaptive controller can be calculated.
Step 4 Substituting the values of $u_{1}$, $u_{2}$ and $u_{3}$ into the Equation (6), the state values of $i_{d}$, $i_{q}$, and ω can be obtained.
Step 5 Determine the accuracy parameter ε. If $\sqrt{{i_{d}}^{2}+{i_{q}}^{2}+\omega^{2}}<\varepsilon$, the PMSM system is considered to have achieved a stable state, then quit, or else return to Step 2.

**Remark** **4.** *When functions $\eta_{1},\eta_{2},\eta_{3},$ are set to zero, system (7) is the same as that of Theorem 1 in [38], which appears to be a deterministic system. The stability theory of ordinary differential equations used in [38] cannot be directly applied to the stochastic PMSM system studied in this paper. Based on the finite-time stability theory of stochastic differential equations, this paper reveals a conclusion contrary to intuition, i.e., that noise perturbation within certain limits can accelerate the realization of stochastic finite-time stability in the PMSM system.*

## 4. Numerical Simulation

In this section, an illustrative example is given to verify the feasibility and effectiveness of the above analytical results. The fourth-order Runge–Kutta method is employed to obtain the numerical solutions. Without losing generality, we set $\eta_{1}\left(i_{d}\right)=\lambda_{1}i_{d}$, $\eta_{2}\left(i_{q}\right)=\lambda_{2}i_{q}$ and $\eta_{3}\left(\omega \right)=\lambda_{3}\omega$, which is also permissible for other linear functions. In this paper, the noise intensity is $\lambda =\lambda_{1}=\lambda_{2}=\lambda_{3}=1.4$, unless otherwise specified. It should be pointed out that it is more difficult to control systems with chaotic phenomena. The system parameters and the initial position are the same as those in [38], where σ=5.46, γ=20, α=7/9, $(i_{d}\left(0\right),i_{q}\left(0\right),\omega \left(0\right))=\left(5,1,-1\right)$. In this paper, we set the tuning parameters $k_{1}\left(0\right)=k_{2}\left(0\right)=k_{3}\left(0\right)=0.4$ and $g_{1}=2,\;g_{2}=1.5,\;g_{3}=2.5.$ To measure the evolution process, we define the time indicator for reaching stability as $K_{0}≜\inf\{t_{1}:∥x\left(t\right)∥<10^{-5},\forall t\geq t_{1}\}$, where $x\left(t\right)={\left(i_{d},i_{q},\omega \right)}^{T}$.

### 4.1. Finite-Time Control of PMSM with Noise Perturbation

**Figure 2:**
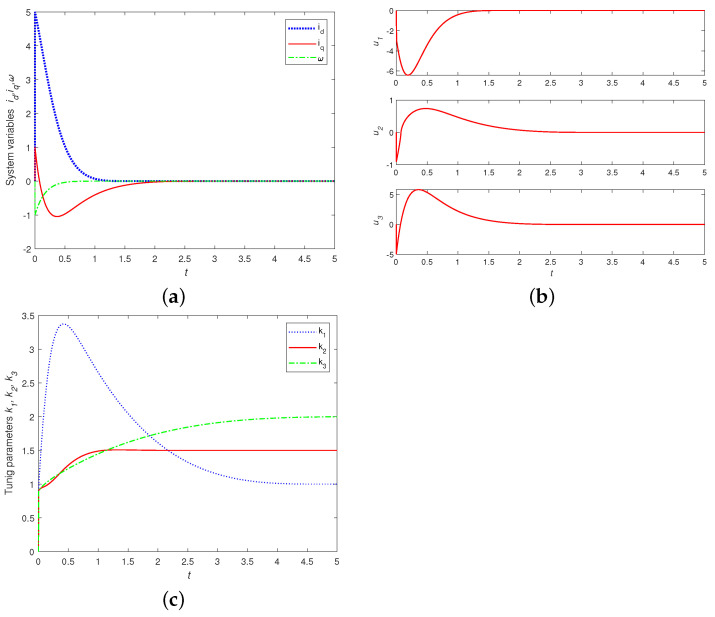
Time responses of: (**a**) controlled state variables $i_{d},i_{q},\omega$; (**b**) adaptive finite-time controllers $u_{1},u_{2},u_{3}$; (**c**) tuning parameters of $k_{1},k_{2},k_{3}$ with $\lambda_{1}=\lambda_{2}=\lambda_{3}=0$ and $i_{d}\left(0\right)=5,\;i_{q}\left(0\right)=1,\;\omega \left(0\right)=-1,\;\sigma =5.46,\;\gamma =20,\;\alpha =7/9$, $g_{1}=2,\;g_{2}=1.5,\;g_{3}=2.5$.

Firstly, we verify the effectiveness of the proposed finite-time control strategy proposed here for the PMSM system without noise perturbation in [38]. Figure 2 displays the time responses of the state variables $i_{d},i_{q}$ and ω, adaptive finite-time controllers $u_{1},u_{2},u_{3}$ and tuning parameters $k_{1},k_{2}$ and $k_{3}$. It is shown that all time responses without noise perturbation are smooth, and the PMSM system can achieve finite-time stability.

**Figure 3:**
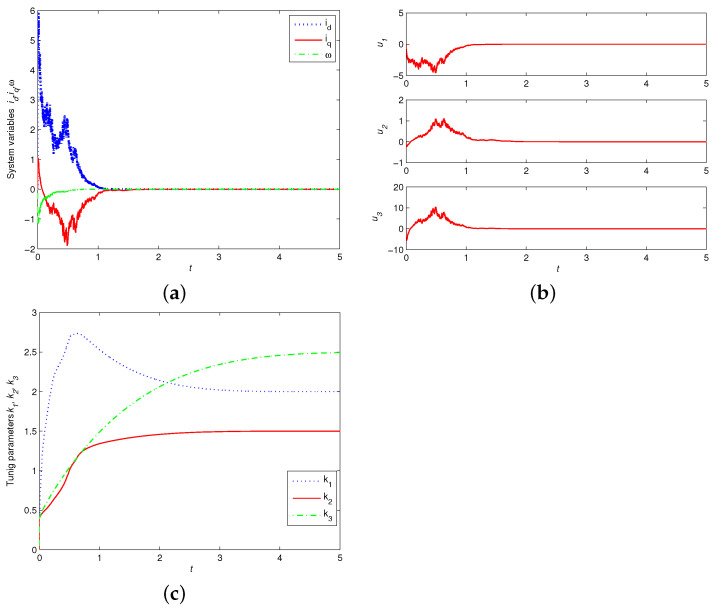
Time responses of: (**a**) controlled state variables $i_{d},i_{q},\omega$; (**b**) adaptive finite-time controllers $u_{1},u_{2},u_{3}$; (**c**) tuning parameters of $k_{1},k_{2},k_{3}$ with $\lambda_{1}=\lambda_{2}=\lambda_{3}=1.4$ and $i_{d}\left(0\right)=5,\;i_{q}\left(0\right)=1,\;\omega \left(0\right)=-1,\;\sigma =5.46,\;\gamma =20,\;\alpha =7/9$, $g_{1}=2,\;g_{2}=1.5,\;g_{3}=2.5$.

Compared with the PMSM without noise perturbation, Figure 3a displays the time responses of the state variables $i_{d},i_{q}$ and ω of the controlled PMSM with noise coupling, which arrives at equilibrium within a finite time by using the proposed adaptive finite-time controllers. It is shown that the undesirable chaos in PMSMs with stochastic noise perturbation can be eliminated effectively within a finite time. Calculated with MATLAB, the time indicator for reaching stability is $K_{0}\approx 2.34.$ Figure 3b shows that the time responses of the adaptive finite-time controllers $u_{1},u_{2},u_{3}$ will settle to zero. Figure 3c displays the time responses of the tuning parameters of terminal attractors $k_{1},k_{2}$ and $k_{3}$, which will converge to the given $g_{1},g_{2},g_{3}$ after the settling time $K_{0}$.

### 4.2. Robust Finite-Time Synchronization and Parameters Identification

**Figure 4:**
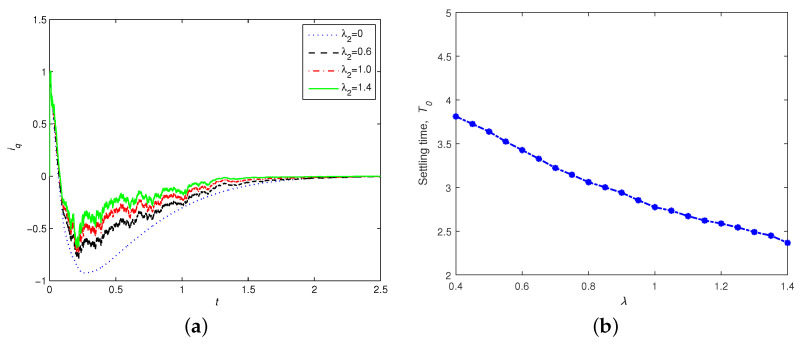
(**a**) Time responses of $i_{q}$ with different noise intensities λ, with $i_{d}\left(0\right)=5,\;i_{q}\left(0\right)=1,\;\omega \left(0\right)=-1,\;\sigma =5.46,\;\gamma =20,\;\alpha =7/9.$ (**b**) Settling time $K_{0}$ as a function of noise intensity λ.

In order to explore the relationship between the convergence time and the noise intensity, we give the time response of representative variables $i_{q}$ with different noise intensities λ=0,0.6,1.0,1.4 in Figure 4a. It is shown that the stronger the noise intensity, the faster the convergence rate. With other system parameters remaining unchanged, we further explored the influence of the noise coupling intensity on the stabilization rate of the system. Figure 4b shows the settling time $K_{0}$ as a function of the control parameter α. With an increase in noise coupling strength, the settling time required for the PMSM system to achieve stochastic finite-time stability is shorter, which means that the PMSM system has good noise robustness under the controller (7).

**Figure 5:**
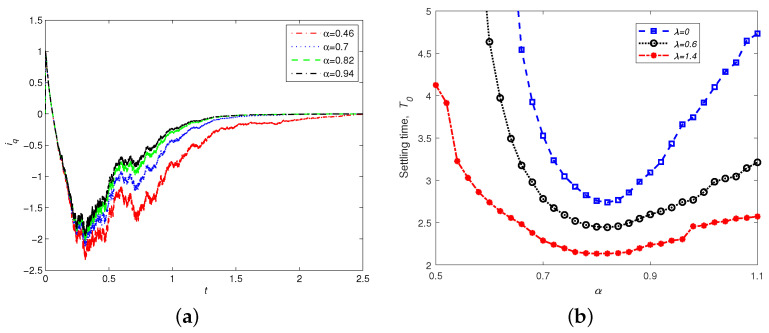
(**a**) Time responses of $i_{q}$ with different control parameters α, with $i_{d}\left(0\right)=5,\;i_{q}\left(0\right)=1,\;\omega \left(0\right)=-1,\;\sigma =5.46,\;\gamma =20,\;\alpha =7/9.$ (**b**) Settling time $K_{0}$ as a function of control parameter α with different λ values.

Remark 2 shows that the convergence time is closely related to the protocol parameter α. To explore the relationship between the convergence time and the control parameter α experimentally, we selected a representative variable $i_{q}$ to demonstrate the convergence. Figure 5a gives the time response of $i_{q}$ with $\lambda =0.6,\;i_{d}\left(0\right)=5,\;i_{q}\left(0\right)=1,\;\omega \left(0\right)=-1,\;\sigma =5.46,\;\gamma =20$ and α=0.46,0.7,0.82,0.94, respectively. It is shown that the convergence rate increases as the parameter α increases. The control parameter α of the adaptive finite-time controller plays a key role in the stability of the PMSM system. Improper selection of control parameters prevents the PMSM achieving finite-time stability. To comprehensively compare the effect of parameters α and λ on the finite-time stability of the PMSM, Figure 5b shows the functional relationship between them. When α=0.81, no matter what value λ takes, the PMSM can achieve finite-time stability in the shortest time. Furthermore, the larger the parameter λ, the smaller the settling time $K_{0}$. However, when α<0.65, the system without noise perturbation (λ=0) cannot achieve stability for the whole operation time. On the one hand, this shows that the finite-time stability of the PMSM depends on the control parameter α, and that noise within certain limits can accelerate the PMSM’s realization of stochastic finite-time stability. On the other hand, $\alpha =\frac{p}{q}<1$ is not a necessary condition for stochastic finite-time stability. When α is slightly larger than 1, the system can also achieve stochastic finite-time stability.

## 5. Conclusions

In this paper, the stochastic finite-time stability of permanent magnet synchronous motors with noise perturbation was investigated. Based on the finite-time stability theory of stochastic differential equations and the adaptive control method, an adaptive finite-time control law was proposed. The effect of the adaptive control parameter α and the noise intensity λ on the PMSM can be observed from numerical simulations. We found that there is an optimal parameter α such that the convergence time is the shortest. Compared to the PMSM without noise perturbation, noise within certain limits can accelerate the PMSM’s realization of stochastic finite-time stability. Since the stochastic settling time is affected by the initial state of the system in this paper, we hope to find a new finite-time control scheme in the future that is independent of the initial state. Another future direction will be to study how to further apply the adaptive finite-time control strategy in practice.

## Data Availability

No applicable.

## Abbreviation

The following abbreviation is used in this manuscript:

PMSM	Permanent Magnet Synchronous Motors
